# Examining Communicative, Critical Health Literacy and eHealth Literacy among International University Students Residing in Japan

**DOI:** 10.3390/healthcare12090941

**Published:** 2024-05-04

**Authors:** Ishtiaq Ahmad, Hira Taimur, Sameera Shabbir, Chaudhry Ahmed Shabbir, Ali Ahsan, Hafiz Sultan Ahmad, Gaku Masuda

**Affiliations:** 1 Department of Global Health Research, Graduate School of Medicine, Juntendo University, Tokyo 113-8421, Japan; 2 Central Campus, International Higher School of Medicine, Bishkek 720054, Kyrgyzstan; 3 Department of Medical Quality and Safety Management, Graduate School of Medicine, Osaka Metropolitan University, Osaka 545-8585, Japan; 4 Department of Biology, Graduate School of Science, Osaka Metropolitan University, Osaka 558-8585, Japan; 5 Faculty of Science and Technology, University of Central Punjab, Lahore 54000, Pakistan; 6 The Section of Global Health, Department of Hygiene and Public Health, Tokyo Women’s Medical University, Tokyo 162-8666, Japan

**Keywords:** health literacy, international students, eHealth literacy, Japan, public health

## Abstract

(1) Background: International students with sufficient health literacy are better equipped to respond to public health emergencies and reduce any unintentional harm that may occur during such events. This study aims to assess the current status of health literacy among international students and investigate the factors that influence health literacy. (2) Methods: A cross-sectional study was conducted in Tokyo on international university students using a questionnaire consisting of the Communicative and Critical Health Literacy and eHealth Literacy Scales. The study analyzed 205 valid responses. Descriptive statistics were utilized to assess the level of health literacy, and linear regression was used to identify the association of socio-demographic characteristics and disease status with health and e-health literacy. (3) Results: Health literacy and e-health literacy were low in 48.29% and 47.29% of international students, respectively. The mean scores of CCHL items ranged from 3.13 to 3.26, while the mean scores of eHEALS items ranged from 3.33 to 3.49. Both health literacy and e-health literacy were better with unmarried status (*p* = 0.015), and e-health literacy was worse with higher age (*p* = 0.007). (4) Conclusions: Overall, international students’ health literacy and e-health literacy were at intermediate levels, with considerable room for improvement, and affected by certain student attributes.

## 1. Introduction

Health literacy, a term introduced in the 1970s, has gained significant importance in public health and healthcare in recent years [1]. Although it is generally regarded as an individual’s ability to understand and use health information to make informed decisions, its definition continues to evolve. Initially, it was defined as the application of fundamental reading, comprehension, and mathematical skills in a healthcare context (AMA Ad Hoc Committee, 1999), while later definitions focused on specific skills that an individual will need to perform healthcare-related tasks [2]. Across all definitions, outcomes are related to the health of individuals, varying only in nuance. A report from the Institute of Medicine mentioned 90 million Americans having difficulty in understanding and acting upon health information, a finding referred to as a “health literacy epidemic” [3]. Around a decade ago, the interest in health literacy was mainly concentrated in the United States and Canada, but now this concept is being increasingly internationalized [4]. The European Commission’s Health Strategy 2008–2013 explicitly mentioned health literacy as an area of priority action [5].

Low health literacy has been shown to be associated with undesirable outcomes such as poor self-rated health [6], higher rates of hospitalization [7], and increased use of emergency services [8]. One suggested pathway between health literacy and health outcomes is via health behaviors such as physical activity [9] and smoking [10]. Rothman et al. showed an association of low health literacy with poor comprehension of food labels [11]. A novel pathway that can connect health literacy to health outcomes could be social connections, since studies have revealed an association between low health literacy and poor communication skills [12,13]. In recent years, the development of e-health literacy has made great contributions to the area of adolescent and adult health literacy, making e-health literacy an important factor in improving health behavior [14]. The internet is one of the key sources to access health-related information, especially for individuals living abroad, who cannot access local information sources due to language barriers and lack of social connections.

The proportion of international students enrolled in Japanese universities is increasing every year, reaching a record number of 310,000 in May 2019 [15]. This trend has led to increased awareness of the various needs and challenges of this population. Apart from the difficulties associated with tertiary education, international students face additional challenges due to relocation, such as lack of social support, language barriers, and financial concerns. Thus, international students may be at increased risk of indulging in health-compromising behaviors as compared to domestic students [16,17]. This population is usually left out of national initiatives aimed at promoting health, preventing diseases, providing treatment and care, and ensuring financial protection for health. The original motivation for this study stems from the recognition that international students, who form a part of the immigrant population in the Japanese context, are often overlooked during social crises such as pandemics. No study has yet explored the health and e-health literacy among the international student population in Japan. This study aims to evaluate the health and e-health literacy status and associated characteristics of this population to support the development of tailored interventions and policies.

## 2. Methods

This cross-sectional study included 205 international university students residing in Tokyo, Japan. The survey was conducted using self-administered questionnaires, employing online Google forms, and online consent was obtained. The questionnaire took approximately 10–20 min to complete.

We employed a combination of purposive and snowball sampling methods to recruit international students for this study. Initially, we used purposive sampling to identify and recruit participants who met our inclusion criteria: international students currently enrolled in universities in Tokyo, Japan. We reached out to international student organizations, university international student offices, and relevant social media groups to promote the study and invite eligible participants. This approach allowed us to target a diverse range of international students from various universities, countries, and fields of study. To expand our reach and increase the diversity of our sample, we also employed snowball sampling. Participants were encouraged to share the study information with their peers and networks who met the inclusion criteria. This method is particularly useful when studying hard-to-reach or dispersed populations, such as international students, as it leverages the social networks of participants to identify additional eligible individuals.

A priori sample size analysis was performed using G*Power 3.1.9.7, based on a two-tailed test, a medium effect size (ρ = 0.3), an α error probability of 0.05, and a power (1-β error probability) of 0.95. The analysis revealed that a sample size of 138 participants would be necessary with 95% power at a 5% significance level.

### 2.1. Outcome Variables

Health literacy and e-health literacy were the two outcome variables. Health literacy was measured by the Communicative and Critical Health Literacy (CCHL) Scale, which has 5 items: the first 3 about communicative health literacy and the last 2 about critical health literacy. Each item is rated on a 5-point Likert scale, from 1 (strongly disagree) to 5 (strongly agree). The average of the raw scores for the five items was calculated to obtain an overall communicative and critical health literacy score for each subject. The theoretical range is 1 to 5. The CCHL Scale is a validated and reliable tool to assess health literacy and has been used in several studies [18,19,20,21,22]. We preferred to use it because it is concise and is based on an established model of health literacy [23]. The internal consistency was adequately high, with the Cronbach’s alpha of the CCHL Scale in this study being 0.95.

Participants’ e-health literacy was measured using the eHealth Literacy Scale (eHEALS) [24], which assesses an individual’s combined knowledge, comfort, and perceived skill in finding, evaluating, and applying their knowledge to improve health issues [24]. This scale consists of 8 items, each measured on a 5-point Likert scale, 1 (strongly disagree) to 5 (strongly agree). Items 1 to 5 assess perceived knowledge, items 6–7 assess skills, and item 8 assesses confidence in finding health information from electronic sources and applying it to the management of health problems. The eHEALS has been validated in many studies among different populations [25,26]. The main reasons for its use are ease of administration and comprehensiveness. The Cronbach’s alpha of eHEALS in our study was 0.96. For the current study, we used English versions of both instruments.

### 2.2. Predictor Variables

Predictor variables were the demographic data, including gender (male/female), age (18–25/26–35/36–45/46–60), marital status (married/unmarried/prefer not to say), study level (undergraduate/postgraduate (master’s)/postgraduate (PhD)) and area of origin (Africa/Asia/Europe/others). The presence of diagnosed non-communicable diseases was also included as a predictor variable. To assess the non-communicable disease status, the participants were asked if they had ever been diagnosed with diabetes or cardiovascular disease in their life. We chose these two chronic diseases because of their relatively high prevalence in the adult population worldwide.

### 2.3. Statistical Analysis

Descriptive analysis was performed, presenting means, standard deviations, frequencies, and percentages, as appropriate. The Shapiro–Wilk test was used to assess the normality of the data. Considering non-normal distributions, the Mann–Whitney U test and Kruskal–Wallis test were used to determine the differences between health literacy and e-health literacy according to gender, age, marital status, study level, area of origin, and disease status (diabetes and cardiovascular disease).

Robust linear regression was conducted to determine the association of health literacy and e-health literacy with demographic data. The Shapiro–Wilk test was used to assess the normality of the distribution of residuals. The Breusch–Pagan test was used to check for homoscedasticity. The variance inflation factor (VIF) was calculated to assess multicollinearity in data.

A *p*-value 0.05 was considered to be statistically significant with a 95% confidence interval. STATA version 15.1 was used for statistical analysis.

## 3. Results

Out of the 205 participants, the majority were males (58.54%). More than half were Asians (57.44%), from countries including China, India, Bangladesh, Pakistan, Malaysia, Korea, Nepal, Philippines, Mongolia, Vietnam, Hong Kong, and Syria; 14.36% of participants were Europeans (from countries like the United Kingdom, Italy, Germany, Spain, and France); 11.28% of participants were of African origin (Gabon, South Africa, Kenya, Nigeria, Namibia, Morocco, and Egypt); and 16.92% came from places other than these three continents (including the US, Canada, Australia, Fiji, Brazil, El Salvador, Jamaica, and New Zealand). Most of the participants were unmarried (62.93%), belonged to the 25–36 years age group (51.22%) and were postgraduate master’s students (54.63%). A small percentage (3.41%) of participants had diagnosed chronic disease (diabetes or cardiovascular). There were significant differences in participants’ health literacy measured by the CCHL Scale according to marital status, area of origin, and disease status. No significant differences were detected in terms of gender, study level, or age. In the case of e-health literacy measured by eHEALS, there were significant differences in terms of participants’ age, marital status, area of origin, and disease status. In terms of gender and study level, the differences were not significant (Table 1).

Previous studies have categorized the sample participants into having low and high health literacy depending on the median of their average CCHL scores [27]. The median score in our sample was 3.2; accordingly, 99 (48.29%) respondents had low health literacy and 106 (51.71%) respondents had high health literacy. All of the statements on the CCHL Scale received mean scores of slightly above 3, indicating satisfactory levels of health literacy in international students overall. Table 2 shows that participants agreeing on items 4 and 5 (which pertain to critical health literacy) clearly outnumbered those disagreeing on those items. Items 1 to 3 (pertaining to communicative health literacy) had roughly same fraction of participants on either side of the neutral response.

The e-health literacy was categorized into low and moderate/high, as in previous studies (keeping a composite score of 24 as a threshold) [28]. More than half of the respondents 107 (52.71%) had moderate/high e-health literacy, while 96 (47.29%) respondents had low e-health literacy. As shown in Table 3, the mean score of each item was above 3, and the sample participants reported fairly good skills in using the internet to obtain, evaluate, and utilize health-related information.

The independent associations of health literacy and e-health literacy were determined using a separate model of linear regression for each scale. For health literacy, the model was significant (R^2^ = 0.191, *p* < 0.001). After adjusting for gender, age, and area of origin, health literacy was significantly better with unmarried status (B = 0.565, 95%CI = 0.113 to 1.018) and in the absence of non-communicable disease (B = 0.820, 95%CI = 0.438 to 1.202) (Table 4).

In the case of e-health literacy, the linear model was significant as well (R^2^ = 0.216, *p* < 0.001). After adjusting for gender and area of origin, e-health literacy significantly worsened for respondents aged between 46 and 60 years (B = −6.790, 95%CI = −11.693 to −1.887) and was better with unmarried status (B = 3.454, CI = 0.090 to 6.817) as well as in the absence of chronic disease (B = 5.950, 95%CI = 1.814 to 10.085) (Table 5).

## 4. Discussion

Health literacy is an essential social determinant of public health [29]. Improvements in health literacy can lead to disease prevention and better health [30], although evidence to clearly prove this point is an area of ongoing research. The post-COVID-19 era has emphasized the importance of health equity and an inclusive approach towards health of populations whereby all components of the society are addressed. The research activities in the area of health literacy in Japan have increased sharply in the last decade [31]; however, the area of health literacy among the international student population in Japan continues to be underexplored. Knowing the level of health literacy among this community is essential to improving the health outcomes of the population living in Japan as a whole. To the best of our knowledge, this is the first study to explore health literacy among international university students residing in Japan. The study provided information on students’ self-perceived competencies in terms of the health and e-health literacy necessary for them to make informed health decisions. The findings of this study add evidence to the limited literature on the health literacy levels of international students in Japan.

In the present study, we assessed health and e-health literacy among international university students residing in Tokyo Prefecture. Students of all ages and study levels were included in the study. Nearly half of the students had low health literacy, and roughly the same proportion had low e-health literacy. This result is consistent with previous studies that assessed health and e-health literacy among students of different countries [32,33,34].

We found age to be a significant predictor of e-health literacy, with higher age (age group 40 to 60) being associated with poor e-health literacy. Thus, older students are lagging behind in e-health literacy skills. This finding is also compatible with previous studies where older students were found to be skeptical about using the internet for obtaining and using health-related information [35,36]. However, this association was not significant in the case of communicative and critical health literacy, confirming the presence of a digital divide and reduced comfort with internet use in older age groups.

Marital status was found to be significantly associated with health literacy and e-health literacy, with unmarried participants showing better levels of both. A similar association was found in a study in Korea, where unmarried men had higher health literacy [37]. This can be attributed to the shift in priorities and increase in responsibilities after marriage, especially while living abroad as students. We did not find any association of level of study with either health or e-health literacy.

We found no difference in either health or e-health literacy in terms of gender. The literature reveals mixed evidence in this area. There is lower health literacy in women in countries where gender disparity prevails, whereas women demonstrate higher health literacy levels in some Western countries [38,39]. Since our sample comprised students from different parts of the world, we could not find any difference in health literacy between male and female respondents.

Our study found significant associations of both health and e-health literacy with chronic disease status. The absence of diagnosed diabetes or cardiovascular disease is associated with better health and e-health literacy. However, due to the very small fraction (3.41%) of students being diagnosed with one of these NCDs, it is difficult to make a valid interpretation of this particular result. Also, due to the cross-sectional design of our study, we could not deduce a causal relationship. Previous studies have explored the mediating role of health-related behaviors to explain this association [40].

This study had some limitations. The data collected were based solely on self-reported measurements, which may have been influenced by response and information bias. Additionally, we used a non-probability sampling method, which has its limitations, such as the potential for bias and the lack of a truly random sample. However, given the exploratory nature of this study and the challenges associated with recruiting a large, representative sample of international students in Japan, we believe that the combination of purposive and snowball sampling was an appropriate and pragmatic approach. To mitigate potential biases, we made efforts to start the snowball sampling from diverse initial contact points, including students from different universities, countries, and fields of study. We also monitored the characteristics of the sample throughout the recruitment process to ensure that we were capturing a range of perspectives and experiences. Another aspect to consider is that we did not take into account the participants’ area of study. It is possible that individuals with a health education background may have influenced the analysis of health literacy levels when comparing health-related students and non-health-related students. The financial situation of the students was also not asked about in the questionnaire, because of the sensitivity of such questions.

While our sample may not be entirely representative of the international student population in Japan, we believe that the diversity of our sample, with participants from various countries, academic levels, and backgrounds, provides valuable insights into the health literacy experiences of this population. Future research could build upon this exploratory study by employing more robust sampling methods, such as stratified random sampling, to obtain a more representative sample.

Based on the research findings, we call upon policymakers and university stakeholders to take proactive measures to address the health literacy needs of international students. These may include developing and implementing culturally sensitive health literacy education programs, expanding multilingual support in university health services, and improving the language accessibility and user-friendliness of online health information resources. By prioritizing these initiatives, we can create a more inclusive and supportive environment that empowers international students to effectively navigate the healthcare system and make informed health decisions.

While this study provides valuable insights into the health literacy of international students in Japan, it also highlights the need for further research in this area. Future studies should consider employing longitudinal designs to investigate the causal relationships between health literacy and health outcomes, conducting qualitative research to gain a deeper understanding of the factors influencing health literacy among international students, and evaluating the effectiveness of interventions designed to improve health literacy in this population. By building upon the foundation laid by this exploratory study, researchers can continue to advance our understanding of health literacy among international students and develop evidence-based strategies to support their health and well-being. By prioritizing the health needs of international students and other marginalized populations, Japan can foster a more inclusive and resilient society, better prepared to handle future public health challenges.

## 5. Conclusions

This study is the first to investigate health literacy among international students in Japan, filling a crucial gap in knowledge within this domain. The findings underscore the importance of understanding and supporting international students’ health literacy, particularly during public health emergencies such as the COVID-19 pandemic, to ensure their well-being and promote health equity.

The results of this study, which reveal limited health literacy and e-health literacy status and significant differences in both levels based on students’ attributes, have important implications for the development of tailored interventions aimed at enhancing health literacy among international students. These insights can guide the design and implementation of targeted education programs, resources, and support services that cater to the diverse needs of this population.

The findings of this study have broader implications for global health, as they contribute to the growing body of knowledge on health literacy among migrant populations. Enhancing health literacy among international students not only promotes health equity but also contributes to the achievement of the United Nations’ Sustainable Development Goals, particularly those related to ensuring healthy lives and promoting well-being for all. By addressing the health literacy needs of this diverse population, we can foster a more inclusive and resilient global community, better prepared to face the health challenges of the future.

## Figures and Tables

**Table 1:** Demographic characteristics of study participants.

Variable	n (%)	Health Literacy			e-Health Literacy		
		Test	z/X^2^ Value	*p*-Value	Test	z/X^2^ Value	*p*-Value
**Gender**		MWU	−0.067	0.945	MWU	0.355	0.722
Male	120 (58.54)						
Female	85 (41.46)						
**Age**		KW	6.671	0.083	KW	13.043	0.004 *
18–25	51 (24.88)						
26–35	105 (51.22)						
36–45	37 (18.05)						
46–60	12 (5.85)						
**Marital status**		KW	11.698	0.002 *		13.632	0.001 *
Married	67 (32.68)						
Unmarried	129 (62.93)						
Prefer not to say	9 (4.39)						
**Study level**		KW	1.876	0.389		3.512	0.172
Undergraduate	69 (33.66)						
Postgraduate (master’s)	112 (54.63)						
Postgraduate (PhD)	24 (11.71)						
**Area of origin**		KW	17.174	0.0007 ***		22.968	0.0001 ***
Africa	22 (11.28)						
Asia	112 (57.44)						
Europe	28 (14.36)						
Others	33 (16.92)						
**Chronic disease**		MWU	−2.813	<0.004 **	MWU	−2.931	<0.003 **
Yes	7 (3.41)						
No	198 (96.59)						

Note: MWU = Mann–Whitney U test, KW = Kruskal–Wallis test; *p*-values: *** *p* < 0.001, ** *p* < 0.01, * *p* < 0.05.

**Table 2:** Health literacy measured by the CCHL Scale.

Item No.	Statement	Strongly Disagree n (%)	Disagree n (%)	Neutral n (%)	Agree n (%)	Strongly Agree n (%)	Item Mean (SD)
1	I can collect health-related information from various sources such as newspapers, books, TV, and the internet.	16 (7.80)	63 (30.73)	35 (17.07)	60 (29.27)	31 (15.12)	3.13 (1.22)
2	I can extract the information needed.	9 (4.39)	69 (33.66)	33 (16.10)	46 (22.44)	48 (23.41)	3.26 (1.26)
3	I can understand and communicate the obtained information.	14 (6.83)	56 (27.32)	39 (19.02)	59 (28.78)	37 (18.05)	3.23 (1.22)
4	I can judge the credibility of the information.	7 (3.41)	55 (26.83)	41 (20.00)	65 (31.71)	37 (18.05)	3.34 (1.15)
5	I can make decisions about plans and actions for improving health based on the information.	14 (6.83)	64 (31.22)	21 (10.24)	65 (31.71)	41 (20.00)	3.26 (1.28)

Scale: 5 = strongly agree, 4 = agree, 3 = neutral, 2 = disagree, 1 = strongly disagree.

**Table 3:** eHealth literacy measured by eHEALS.

Item No.	Statement	Strongly Disagree n (%)	Disagree n (%)	Neutral n (%)	Agree n (%)	Strongly Agree n (%)	Item Mean (SD)
1	I know what health resources are available on the Internet	15 (7.32)	54 (26.34)	38 (18.54)	43 (20.98)	55 (26.83)	3.33 (1.31)
2	I know where to find helpful health resources on the Internet	7 (3.41)	58 (28.29)	39 (19.02)	66 (32.20)	35 (17.07)	3.31 (1.15)
3	I know how to find helpful health resources on the Internet	9 (4.39)	61 (29.76)	35 (17.07)	58 (28.29)	42 (20.49)	3.30 (1.22)
4	I know how to use the Internet to answer my questions about health	15 (7.32)	72 (35.12)	20 (9.76)	56 (27.32)	42 (20.49)	3.18 (1.30)
5	I know how to use the health information I find on the Internet to help me	9 (4.41)	61 (29.90)	31 (15.20)	47 (23.04)	56 (27.45)	3.39 (1.28)
6	I have the skills I need to evaluate the health resources I find on the Internet	6 (2.93)	56 (27.32)	37 (18.05)	66 (32.20)	40 (19.51)	3.38 (1.16)
7	I can tell high quality health resources from low quality health resources on the Internet	6 (2.93)	46 (22.44)	43 (20.98)	60 (29.27)	50 (24.39)	3.49 (1.16)
8	I feel confident in using information from the Internet to make health decisions	8 (3.92)	42 (20.59)	42 (20.59)	65 (31.86)	47(23.04)	3.49 (1.16)

Scale: 5 = strongly agree, 4 = agree, 3 = neutral, 2 = disagree, 1 = strongly disagree.

**Table 4:** Predictors of health literacy.

Variable	Coefficient	95% CI	*p*-Value
**Marital status**			
Married	1 Ref		
Unmarried	0.646	0.197 to 1.095	0.005 **
Prefer not to say	0.437	−0.275 to 1.151	0.228
**Study level**			
Undergraduate	1 Ref		
Postgraduate (master’s)	−0.077	−0.414 to 0.259	0.652
Postgraduate (PhD)	−0.211	−0.833 to 0.409	0.502
**NCD**			
Yes	1 Ref		
No	0.725	0.341 to 1.110	<0.001 ***

Note: Linear regression model adjusted for gender, age, and area of origin. R^2^ = 0.243, CI = confidence interval; *p*-values: *** *p* < 0.001, ** *p* < 0.01.

**Table 5:** Predictors of e-Health literacy.

Variable	Coefficient	95% CI	*p*-Value
**Age**			
18–25	1 Ref		
26–35	−1.463	−4.590 to 1.663	0.357
36–45	0.524	−3.988 to 5.037	0.819
46–60	−6.329	−11.001 to −1.657	0.008 **
**Marital status**			
Married	1 Ref		
Unmarried	3.454	0.090 to 6.817	0.044 *
Prefer not to say	2.085	−3.310 to 7.481	0.447
**Study level**			
Undergraduate	1 Ref		
Postgraduate (master’s)	−1.295	−3.888 to 1.296	0.325
Postgraduate (PhD)	−2.680	−7.156 to 1.794	0.239
**NCD**			
Yes	1 Ref		
No	5.377	1.188 to 9.567	0.012 *

Note: Linear regression model adjusted for gender and area of origin. R^2^ = 0.250, CI = confidence interval; *p*-values: ** *p* < 0.01, * *p* < 0.05.

## Data Availability

Data are contained within the article.
